# New targeted molecular therapies for cancer: radiological response in intrathoracic malignancies and cardiopulmonary toxicity: what the radiologist needs to know

**DOI:** 10.1186/1470-7330-14-26

**Published:** 2014-07-23

**Authors:** Frederico F Souza, Andrew Smith, Cyrillo Araujo, Jyothi Jagannathan, Ciaran Johnston, Kevin O’Regan, Atul Shinagare, Nikhil Ramaiya

**Affiliations:** 1 Department of Radiology, University Of Mississippi Medical Center, 2500 North State Street, Jackson, MS, USA; 2 Department of Imaging And Nuclear Medicine, Dana-Farber Cancer Institute, Harvard Medical School, 44 Binney Street, Boston, MA, USA

**Keywords:** Molecular targeted therapies, Tumor response, Computed tomography

## Abstract

The emergence of new novel therapeutic agents which directly target molecules that are uniquely or abnormally expressed in cancer cells (molecular targeted therapy, MTT) has changed dramatically the treatment of cancer in recent years. The clinical benefit associated with these agents is typically limited to a subset of treated patients, who in many cases are defined by a specific genomic mutations and expression lesion within their tumor cells. All these new therapy modalities represent new challenges to radiologists as their mechanism of action and side effect profiles differ from conventional chemotherapy agents. In this article we will discuss radiological patterns of response to molecular targeted therapies MTT in lung cancer, typical and atypical radiological responses of targeted molecular therapy for other intra thoracic malignancies, cardiopulmonary toxicity and other side effects of molecular targeted therapy MTT in the thorax.

## Introduction

The emergence of novel therapeutic agents that directly target molecules that are uniquely or abnormally expressed in cancer cells (molecular targeted therapy, MTT) has changed dramatically the treatment of cancer in recent years [1]. The clinical benefit associated with these agents is typically limited to a subset of treated patients, who in many cases are defined by specific genomic mutations and expression within their tumor cells. All these new therapy modalities represent new challenges to radiologists as their mechanism of action and side effect profiles differ from conventional chemotherapy agents [2]. In this article we will discuss radiological patterns of response to MTT in lung cancer, typical and atypical radiological responses of targeted molecular therapy for other intrathoracic malignancies, cardiopulmonary toxicity and other side effects of MTT in the thorax.

### New concepts in molecular targeted therapy

The role of MTT is to reduce or inhibit proliferative activity in cancer cells and block intracellular signaling pathways, blocking specific enzymes responsible for cancer growth and proliferation. Among these important MTT agents approved by the US Food and Drug Administration (FDA) are imatinib mesylate (Gleevec®), approved to treat gastrointestinal stromal tumor, trastuzumab (Herceptin®), approved to treat certain types of breast cancer as well as some types of gastric or gastroesophageal junction adenocarcinomas, and everolimus (Afinitor®), approved to treat patients with advanced kidney cancer whose disease has progressed after treatment with other therapies. In the highly vascular metastatic tumors hepatocellular carcinoma (HCC) and renal cell carcinoma (RCC), successful response to anti-angiogenic therapy has been associated with the use of sunitinib (Sutent®) and sorafenib (Nexavar®), respectively. The response is assessed by decreased tumor size, decreased tumor attenuation, and tumor necrosis on the post-therapy contrast-enhanced computed tomography (CT) studies [3].

### Molecular targeted therapy for lung cancer

First-line chemotherapy for lung cancer often includes a platinum-based drug (cisplatin or carboplatin) in combination with another FDA-approved chemotherapy drug (paclitaxel, docetaxel, etoposide, gemcitabine, pemetrexed) [3]. However, in a subset of patients with non-small-cell-lung cancer (NSCLC), there is overexpression of epidermal growth factor receptor (EGFR). Stimulation of the EGFR pathway leads to a series of intracellular events culminating in increased mitotic and growth potential, increased ability to metastasize, and increased angiogenesis (new blood vessel formation) in the cancer cells.

Many factors that correlate with favorable response occur in patients with particular clinical characteristics, such as a higher frequency of EGFR mutations (which themselves appear to be closely associated with higher likelihood of response to EGFR inhibitors) among Asians vs. non-Asians, women vs. men, never-smokers vs. current or prior smokers, and/or patients with adenocarcinomas vs. squamous histology tumors [4]. New developments in the management of NSCLC include more aggressive surgical techniques, the use of neoadjuvant chemoradiation prior to surgery and use of molecular targeted therapeutic agents [4-7]. The MTT agents currently FDA approved for lung cancer are gefitinib and erlotinib. These MTT agents have shown efficacy in first and second-line treatment regimens as monotherapy or in combination with conventional chemotherapy agents [7].

### Radiological assessment of response to treatment in cancer

Radiological assessment of response to treatment in lung cancer can be further divided into typical and atypical patterns of response. Typical patterns of response include: A) decrease in tumor size, B) decrease in vascularity (e.g. anti-angiogenic agent effect), C) presence of cavitary changes within the mass, and D) decrease in metabolism when F­18­fluorodeoxyglucose (FDG)- positron emission tomography (PET)/CT is used to evaluate treatment response. Atypical patterns of response include: A) increase in the size of a mass with decreased tracer uptake, B) presence of intralesional and/or perilesional hemorrhage with stable or increased size of the mass.

## Review

### Typical response patterns

**Figure 1:**
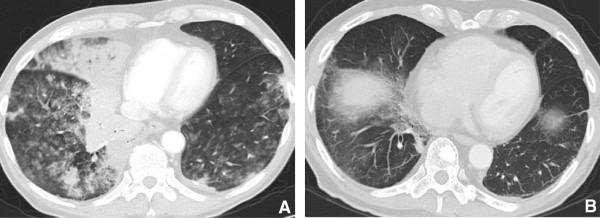
**77 year old male with multifocal bronchoalveolar cell carcinoma on erlotinib (Tarceva) showing marked interval response to therapy with decrease in size of right peri hilar mass. A**. Infiltrative consolidative opacity involving the peri hilar region and the medial aspect of the right lower lobe. **B**. After 2 cycles of therapy with Tarceva there is significant reduction in the tumor burden with minimal residual opacity in the medial aspect of the right lower lobe. Fibrosis due to post treatment changes in the RLL.

**Figure 2:**
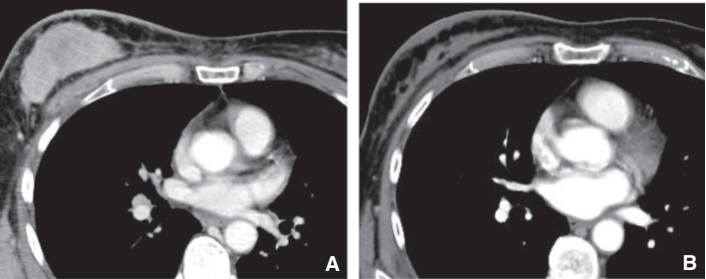
**65 year old female with NSCLC and metastatic breast mass. A**. Large heterogeneous soft tissue mass in the right breast is seen consistent with metastatic NSCLC. **B**. After 4 cycles of chemotherapy with Tarceva there is significant decrease in the size of the metastatic mass in the right breast.

**Table 1:** Tumor response criteria — WHO, RECIST 1.0 and RECIST 1.1 and classification of tumor assessment

**WHO**	**RECIST 1.0**	**RECIST 1.1**
No particular number of lesions specified	Requires 10 targets per five organs when measuring the tumor burden	Requires only five targets (two per organ).
Bi dimensionally measurable lesion; no stipulation of minimal size of the lesion	Measures the long axis of lymph nodes as for other lesions	Measures the short axis of lymph nodes and long axis for other lesions
50% decrease in target lesions, without a 25% increase in any one target lesion; confirmed at 4 wk	Defines progression as a 20% increase in sum	A 20% increase and at least 5 mm absolute increase.
	States that disease progression for non-measurable disease “must be unequivocal”	Expands that definition to convey an impact on the overall burden of disease.
	States that confirmation is required but in new version only if response is primary endpoint	Includes a new section that incorporates a comment on FDG PET interpretation

**Figure 3:**
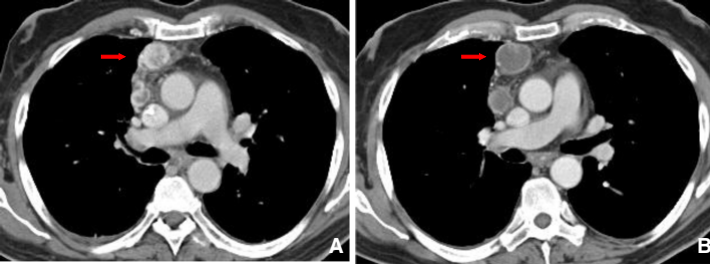
**68 year old male with metastatic thyroid cancer.** CT scan with contrast (Figure **A**) shows hyper vascular mediastinal nodes (arrow) consistent with metastatic disease. CT scan obtained after 2 cycles of Gefitinib (Figure **B**) demonstrates decrease in the attenuation of mediastinal nodes (arrow) reflecting desvascularization, with a thin rim of residual peripheral enhancement.

**Figure 4:**
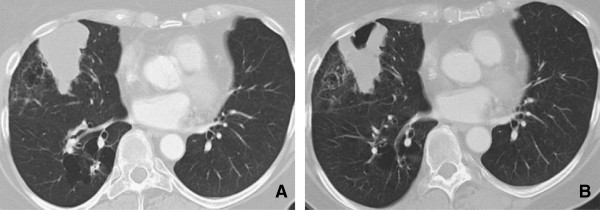
**65 year old female with NSCLC.** Initial CT scan shows large soft tissue mass in the RML (Figure 4**A**). After 3 cycles of Bevacizumab (Avastin) the mass demonstrates typical antiangiogenesis effect with peripheral and cavitation associated with mild overall decrease in the size of the mass (Figure 4**B**).

**Figure 5:**
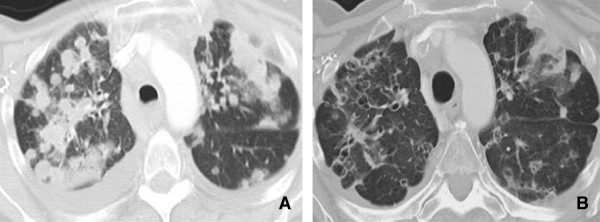
**58 year old female with metastatic breast cancer. A**. demonstrates multiple metastatic pulmonary nodules in both lungs. **B**. After 3 months of bevacizumab (Avastin) therapy there has been remarkable decrease in size and cystic change in lung metastases.

**Figure 6:**
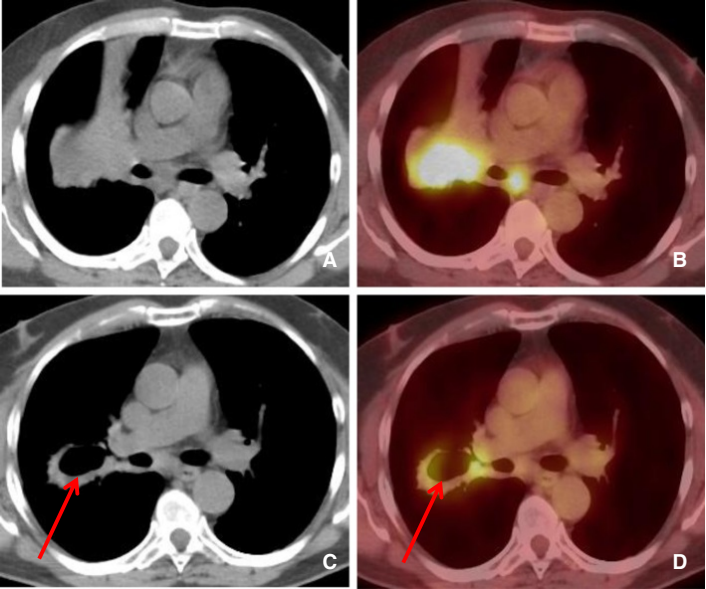
**56 year old female with NSCLC treated with Bevacizumab. A-B**. Computed tomography and PET-CT demonstrating soft tissue mass in the right middle lobe extending to the right hilar region demonstrating significantly increased tracer uptake. **C-D**. After 2 cycles of Bevacizumab (Avastin) there has been interval development of pulmonary cavitation within the central portion of the RML mass. PET-CT showing central cavitation and decrease in FDG uptake in the central portion of the mass (arrow). Persistent peripheral soft tissue and right hilar adenopathy are seen.

A. Decrease in tumor size

Histopathology is often used as the reference standard for assessing the response to primary chemotherapy in lung cancer. However, there is no single definition of a histopathologic response, and response criteria vary among studies. Most commonly, a pathologic complete response is defined by the absence of residual invasive tumor [8,9]. Other response classifications include changes in tumor cellularity and the presence of regressive changes in residual tumor tissue [10] (Figures 1 and 2).

In radiology, is generally accepted that a decrease in tumor size correlates with treatment effect; as a result, imaging was adopted for lesion measurement in the World Health Organization (WHO) criteria in 1979. However, because of some limitations of the WHO criteria, the Response Evaluation Criteria in Solid Tumors (RECIST) was introduced in 2000. Subsequently in October 2008 at the 20th EORTC-NCI-AACR Symposium on Molecular Targets and Cancer Therapeutics (Geneva, October 2008) a New Response Evaluation Criteria in Solid Tumors: Revised RECIST Guidelines (version 1.1) was adopted [Table 1].

Both WHO and RECIST make use of anatomic imaging, predominantly CT, to obtain measurements of reference tumor lesions before and after treatment for response assessment and follow-up. However, functional imaging such as dynamic contrast-enhanced magnetic resonance imaging (DCE-MRI), perfusion CT and PET imaging have been found to be more useful than morphological changes in the assessment of tumor response [11]. Goudarzi et al [12]. in their retrospective analysis of 630 patients with biopsy proven lung cancer demonstrated that patients with pure bronchoalveolar carcinoma (BAC) exhibit a lower FDG uptake and lower tumor attenuation compared with those with adenocarcinoma.

B. Decrease in tumor vascularity

Tumor vascularity can be assessed by measuring blood flow kinetics using dynamic contrast enhancement either with dual energy CT or perfusional MRI. Subjective evaluation using changes in tumor attenuation and tumor vascularization, in addition to changes in tumor size, is considered a good way to evaluate response by computed tomography [13] (Figure 3). Measuring Hounsfield units can serve as a surrogate of tumor vascularity as tumors decrease in density due to necrosis as they respond to therapy. Early clinical trials have indicated, however, that conventional imaging strategies that use tumor size or other structural criteria may not be suitable for monitoring the effects of anti-angiogenesis drugs [14]. An effective imaging strategy for assessing tumor vascularity would also be of value to monitor “anti-angiogenesis” drugs that aim to halt cancer progression by suppressing the tumor blood supply. Perfusion CT is potentially well suited to monitoring tumor response to anti-angiogenesis agents [15]. Dynamic enhanced CT techniques using quantitative enhancement parameters may provide a tool to evaluate the heterogeneity of tumor vascularity and angiogenesis and CT measurements of perfusion have been shown to be reproducible and have been validated against a range of reference methods [15-25].

C. Cavitation

Cavitation has been commonly associated with squamous subtypes and can be seen occasionally in adenocarcinoma. Bevacizumab (Avastin) is a humanized monoclonal antibody against vascular endothelial growth factor (VEGF) that has been associated with cavitation in primary and metastatic lung cancer. Trials of VEGF inhibitors have shown that responding lesions frequently exhibit marked central cavitation [26]. It has been postulated that cavitation occurs through central necrosis of lesions after inhibition of angiogenesis. Marom et al [27] described a retrospective single-institution experience of 124 patients treated with various angiogenesis inhibitors in which 14% of patients developed cavitation. A potential link between clinically relevant pulmonary hemorrhage and cavitation has been raised by studies of bevacizumab warrants further investigation, however (Figures 4 and 5).

D. Decreased metabolism

Frequently, there is a discrepancy between the anatomic appearance and the metabolic activity of the tumor during evaluation of response to anti-angiogenic drugs. Changes in tumor size do not necessarily correlate with changes in tumor viability and outcome [28]. Therefore, it might be more appropriate to assess tumor response by metabolic activity, which can be measured by FDG uptake on PET or [18 F] FDG PET/CT. Decrease in FDG uptake after treatment may prove to be a better indicator of a favorable response rather than change in tumor size (Figure 6).

Hicks et al [28] evaluated aggregated data on the use of 18 F-FDG PET and PET/CT in therapeutic response assessment in NSCLC and the data strongly indicated that a reduction in tissue 18 F-FDG uptake, measured at whatever time after treatment, is more likely to be associated with both a pathologic response and improved survival than when there was no evidence of decreased uptake by the tissue.

### Atypical response patterns

A. Increase in the size of a mass with decreased metabolism

The overall increase in size and decrease in tracer uptake in a mass is a phenomenon that has been extensively described in gastrointestinal stromal tumor treated with imatinib mesylate (Gleevec), reflecting therapy response [13]. In our series we also observed similar findings in patients with lung cancer. Once the patient is treated, the tumor usually decreases in size upon response. In some responding tumors, the tumor size increases as a result of intratumoral and perilesional hemorrhage, necrosis, or myxoid degeneration. The attenuation of the tumor decreases significantly, as the tumor becomes homogeneous.

B. Presence of intralesional and/or perilesional hemorrhage with stable or increased size of the mass.

### Challenges and future directions

The discovery of the correlation of EGFR mutations and response to EGFR TKI therapy has demonstrated that molecular typing of tumors to guide therapy selection for lung cancer is possible. Prospective trials will need to validate the feasibility and efficacy of selecting therapy based on tumor molecular profiles. Also, numerous molecularly targeted agents are in clinical development, and future studies will need to identify molecular and clinical tools to guide the use of these agents, particularly in combination with current therapies such as surgery, radiation, cytotoxic chemotherapy, and other targeted therapies.

#### The role of computed tomography-guided core-needle biopsy in demonstrating epidermal growth factor receptor mutations in patients with non-small-cell lung cancer

The use of surgical specimens to identify EGFR mutations obtained is invasive, expensive, and increases morbidity and mortality. Alternatively, prior reports on EGFR mutation analysis in nonsurgical materials such as biopsy specimens, pleural effusions, and serum have noted problems of contamination by non-target normal cells within the samples [29-32]. CT-guided coaxial core-needle biopsy of the lung has been proven to have high diagnostic accuracy, sensitivity, specificity, and negative predictive values in advanced NSCLC and enables the acquisition of sufficient tissue for EGFR gene mutation analysis [33].

#### Pulmonary toxicity from novel antineoplastic agents

Antineoplastic agent-induced pulmonary toxicity is complex and a diagnosis of exclusion [34]. Similarly, the pathogenesis of antineoplastic agent-induced lung injury is poorly understood and several mechanisms have been suggested, including direct injury to pneumocytes (chemical alveolitis) or the alveolar capillary endothelium and the subsequent release of cytokines and recruitment of inflammatory cells [35-37].

Other causes of respiratory failure, including pneumonia, cardiogenic pulmonary edema, and diffuse alveolar hemorrhage, should be excluded. These conditions are not easily differentiated based on clinical presentation and radiographic findings. Furthermore, as patients usually receive multiple antineoplastic agents, it is usually difficult to identify the culprit agent.

The clinical and radiologic manifestations of these drugs generally reflect the underlying histopathologic processes and include diffuse alveolar damage (DAD), interstitial pneumonitis, cryptogenic organizing pneumonia (COP), eosinophilic pneumonia, obliterative bronchiolitis, pulmonary hemorrhage, edema, hypertension, or veno-occlusive disease. Intra-thoracic and extra pulmonary side effects of these drugs include skin thickening, pleural effusions and thromboembolism.

#### Diffuse alveolar damage

DAD is a common manifestation of drug-induced lung injury that results from necrosis of type II pneumocytes and alveolar endothelial cells [35]. DAD is divided into an acute exudative phase and a late reparative or proliferative phase.

**Figure 7:**
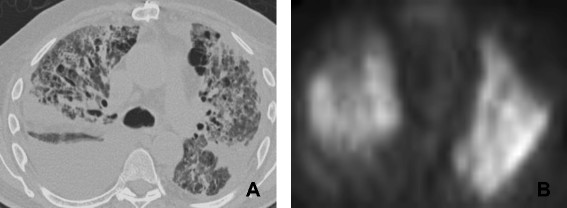
**66 year old male with post transplant lymphoproliferative disorder on rituximab. A**. CT scan of the chest shows diffuse abnormality with bilateral effusions, ground glass attenuation, thickened interstitium and coarse linear parenchymal bands. **B**. PET image shows diffuse parenchymal uptake of FDG in keeping with a diffuse inflammatory process. Biopsy revealed diffuse alveolar damage.

Drugs that may cause DAD include gefitinib, an oral EGFR tyrosine kinase inhibitor that is active against NSCLC, ovarian, colon, head and neck, and breast cancers. Gefitinib-induced lung toxicity usually occurs within the first 90 days of treatment. Interstitial pneumonitis, alveolar hemorrhage, and pulmonary fibrosis have been described [38-43].Chest radiographs show bilateral heterogeneous or homogeneous opacities, often distributed in the mid and lower lung. High-resolution CT in early DAD typically shows scattered or diffuse areas of ground-glass opacity. When progressive fibrosis develops, marked architectural distortion and honeycomb lung can occur (Figure 7).

#### Interstitial pneumonitis

Interstitial pneumonitis is characterized by areas of scattered expansion of the interstitium by inflammatory cells, mild interstitial fibrosis, and reactive hyperplastic type II pneumocytes [35]. New targeted molecular therapies for lung cancer that can cause interstitial pneumonitis include: erlotinib, trastuzumab, temsirolimus, everolimus and gefitinib.

Erlotinib (Tarceva) is indicated for the treatment of patients with locally advanced or metastatic NSCLC. Vahid and Esmaili [44] described two cases of erlotinib-induced pneumonitis that resulted in respiratory failure. The patients presented 4 to 6 days after the initiation of erlotinib therapy with fever, cough, and hypoxemia.

Trastuzumab is a humanized monoclonal antibody that selectively binds to the human EGFR (HER)-2 protein, indicated for the treatment of metastatic breast cancers with over expression of HER-2 protein. The incidence of trastuzumab-induced pneumonitis is 0.4 to 0.6%. Trastuzumab-induced pneumonitis may present with rapidly progressive pulmonary infiltrates and respiratory failure. Infusion-related symptoms, including hypotension, angioedema, bronchospasm, dyspnea, fever, chills, and urticaria, have been reported to occur in about 15% of patients. Severe episodes of hypotension, bronchospasm, and hypoxemia were also observed [45-49].

Temsirolimus is a rapamycin analog that is active against renal cell carcinoma, endometrial carcinoma, breast cancer, glioblastoma multiforme, and gastrointestinal neuroendocrine tumors. Interstitial pneumonitis is a non–dose-dependent complication of temsirolimus. Interstitial pneumonitis has been reported in 1 to 36% of patients and the onset of pneumonitis usually takes place within 16 weeks (range 2 to 16 weeks) after temsirolimus treatment [50,51].

Everolimus is an mTOR inhibitor that has been used as an investigational antineoplastic agent (e.g. for the treatment of sarcoma or renal cell cancer). Although clinical data in patients with malignancy are sparse, there have been previous reports of interstitial pneumonitis with everolimus in heart transplant recipients [52].

Rituximab was first approved for the treatment of low-grade follicular lymphoma, and is now also approved for high-grade lymphomas, while it has also been used in other hematological diseases. The mechanism of action targets CD20+ B lymphocytes. Although rituximab-induced lung injury is rare, it has been well documented [53-56].

Interstitial pneumonitis has been reported with rituximab monotherapy, thus proving the potential of this agent for lung injury. In all cases lung toxicity resolved after steroid treatment, with no late sequelae. Nevertheless, it should be mentioned that concomitant administration of steroids may not prevent the occurrence of pneumonitis [53].

**Figure 8:**
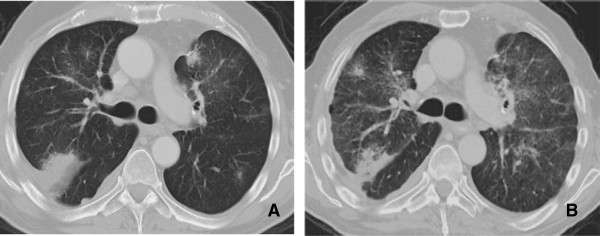
**Interstitial Pneumonitis 58 year old male with NSCLC on bevacizumab. A**: Pre treatment images demonstrate consolidative mass in the posterior segment of the right upper lobe. **B**: Post treatment CT shows marked reduction in size of a posterior segment RUL mass but with interval development of ground glass attenuation, interstitial thickening and parenchymal bands. Biopsy was performed and showed Interstitial pneumonitis.

**Figure 9:**
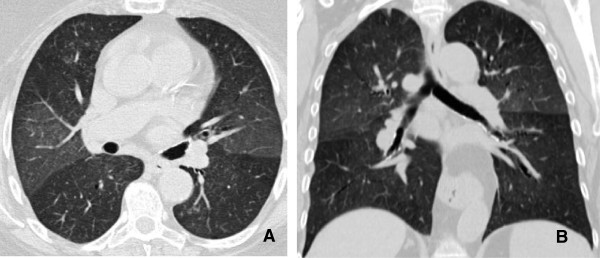
**Interstitial pneumonitis. A** and **B**: Axial and Coronal CT scan of the chest demonstrates diffuse areas of ground glass opacity predominantly involving the upper lobes associated with centrilobular nodules. There has been sparing of the lower lobes consistent with Rituximab induced interstitial pneumonitis.

Radiological findings on chest radiographs in interstitial pneumonitis usually show diffuse heterogeneous opacities throughout both lungs. Areas of consolidation may also be present [57] (Figure 8). Early high-resolution CT scans may show only scattered or diffuse areas of ground-glass opacity and centrilobular opacities (Figure 9) [58]. Later, findings of fibrosis (traction bronchiectasis, honeycombing) predominate in a basal distribution.

#### Cryptogenic organizing pneumonia

COP is a nonspecific histopathologic pattern of lung injury characterized by the proliferation of immature fibroblastic plugs within the respiratory bronchioles, alveolar ducts, and adjacent alveolar spaces [59]. Affected patients present with progressive dyspnea, dry cough, and fever.

Trastuzumab treatment has been reported to be a causative agent of COP. Infusion-related symptoms, including hypotension, angioedema, bronchospasm, dyspnea, fever, chills, and urticaria, have been reported to occur in about 15% of patients.

**Figure 10:**
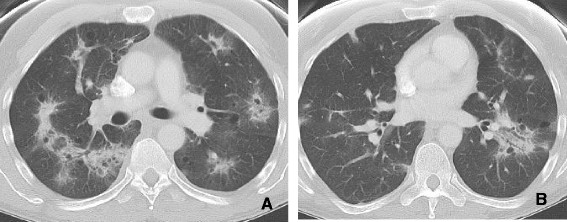
**COP. A** and **B**: CT scan shows associated poorly defined nodular areas of consolidation and cavitation along the bronchovascular bundle. Presence of centrilobular nodules and associated bronchial dilatation. Findings are consistent with COP (Cryptogenic organizing pneumonia).

Chest radiographs demonstrate bilateral scattered heterogeneous and homogeneous opacities, which are typically peripheral in distribution and are equally distributed between the upper and lower lobes. CT often shows associated poorly defined nodular areas of consolidation, centrilobular nodules and “tree-in-bud opacities”, and bronchial dilatation [59]. The pattern of parenchymal opacity usually follows the bronchovascular bundle (Figure 10). Patients with COP typically respond well to cessation of drug therapy, but the patient may also require the administration of corticosteroids to improve the radiological and clinical picture.

#### Eosinophilic pneumonia

**Figure 11:**
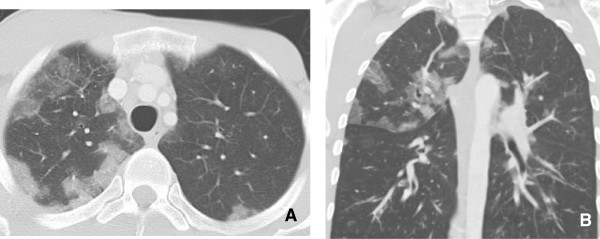
**Eosiniphilic pneumonia. A** and **B**. Thin-section (1-mm collimation) CT scan (lung windowing) shows multifocal patchy areas of ground-glass opacity and consolidation predominantly located in the periphery of upper lobes. Findings are consistent with Eosiniphilic pneumonia.

Eosinophilic pneumonia is characterized by alveolar septal thickening and infiltration by eosinophils, lymphocytes, and plasma cells [60]. Causative drugs are trastuzumab and oxaliplatin. Affected patients typically present with progressive dyspnea, dry cough, and occasionally fever. Peripheral eosinophilia and elevated immunoglobulin E levels are common.Chest radiographs show homogeneous opacities that typically have a peripheral and upper lobe distribution with sparing of the central portions of the lung (Figure 11). CT can be useful for demonstrating the peripheral nature of the pulmonary opacities. Patients with eosinophilic pneumonia caused by drug therapy usually respond well to cessation of the therapy but the administration of corticosteroids may also be required.

#### Pulmonary hemorrhage

**Figure 12:**
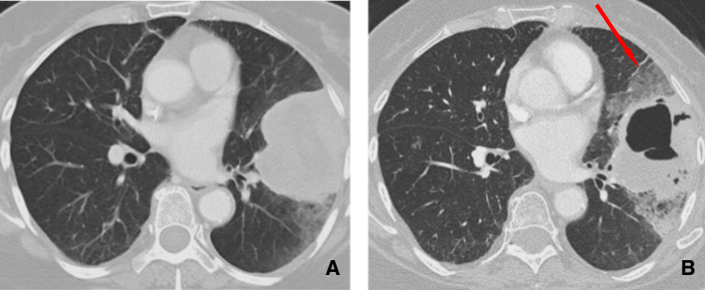
**71 year old female with NSCLC on an erlotinib. A**: Initial CT shows a large and lobulated left upper lobe mass extending to the pleural surface. **B**: CT scan obtained 2 months later demonstrates cavitation within the central portion of the mass with development of surrounding ground glass opacities secondary to intralveolar hemorrhage (arrow).

Diffuse pulmonary hemorrhage is an uncommon complication with former chemotherapy regimens, but has become more common with the introduction of new MTT and anti-angiogenic agents. Pulmonary hemorrhage and hemoptysis have been reported in 2.3% of patients with nonsquamous NSCLC on bevacizumab (Avastin) therapy [61-63]. Severe hemoptysis and pulmonary hemorrhage associated with bevacizumab therapy is more common in patients, with squamous cell carcinoma being reported in up to 31% of patients.Chest radiographs typically show bilateral heterogeneous and homogenous opacities. Focal consolidation is a less common finding. High-resolution CT usually shows scattered or diffuse areas of ground-glass opacity with intralobular septal thickening (crazy paving pattern), which can mimic atypical infection and edema (Figure 12).

#### Pulmonary edema and fluid retention

The pathogenesis of antineoplastic agent-induced pulmonary edema is poorly understood and has been suggested to be due to direct injury to pneumocytes (chemical alveolitis) or the alveolar capillary endothelium and the subsequent release of cytokines, which induce pulmonary edema due to leaky capillaries. Drugs that may lead to increased permeability and subsequent edema include imatinib, dasatinib and rituximab.

Imatinib is a potent tyrosine kinase inhibitor, which is mainly used in the treatment of chronic myelogenous leukemia (CML) and is also effective in patients with gastrointestinal stromal tumor (GIST). Although most cases of imatinib-induced pulmonary adverse events have been reported in patients with CML, there have been cases of dyspnea during imatinib therapy, most often related to fluid retention and pulmonary edema [64].

**Figure 13:**
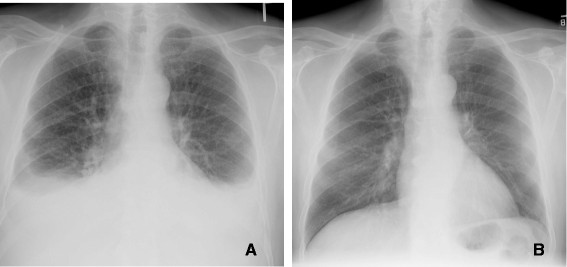
**Dasatinib induced effusion. A**. 72 year old male with CML with disease breakthrough on imatinib, 2 weeks after the switch to dasatinib. PA CXR shows bilateral effusions. **B**. After dose reduction 1 month later, there was interval resolution of bilateral pleural effusions.

**Figure 14:**
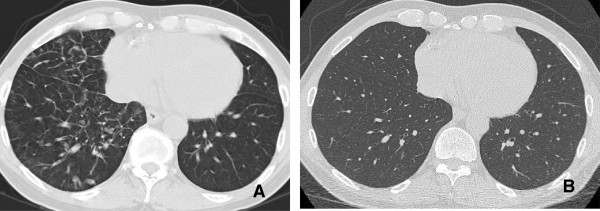
**Imatinib induced pulmonary edema. A**. CT scan on the chest demonstartes thickening of the interlobular septa in the right lung base with no evidence of pleural effusion. **B**. Restaging CT scan obtained 1 month later shows resolution of the pulmonary edema after dose reduction.

Dasatinib is a potent inhibitor of SRC/ABL kinase inhibitor used in the treatment of CML in patients who had disease resistant to or who could not tolerate imatinib [65]. A study by Kantarjian et al [66]. compared dasatinib to imatinib and demonstrated that superficial edema (42% versus 15%) and fluid retention (45% versus 30%) were more common in the imatinib group, while pleural effusion (Figure 13) was more common with dasatinib (17% versus 0%).Characteristic findings of pulmonary edema on chest radiographs include pulmonary vascular redistribution, poor definition of pulmonary vascular markings, septal (Kerley B) lines, and pleural effusions that may or may not be associated with cardiomegaly (Figure 14). On high-resolution CT, findings of patchy areas of ground-glass opacity, combined with consolidation and intra- and interlobular interstitial thickening, are seen in the majority of patients with pulmonary edema.

#### Effusions

Pulmonary complications are uncommon and are usually associated with a fluid retention syndrome. The manifestations from the lungs are pleural effusions and pulmonary edema [67,68] seen in patients treated with imatinib and dasatinib. It seems that these drugs are associated with rare but well documented lung toxicity and this should be taken into consideration in the differential diagnosis of otherwise unexplained pleural effusions.

#### Thromboembolic manifestations

VEGF antagonism could cause decreased matrix deposition in the supporting layers of vessels; therefore, the final picture of anti-VEGF therapy might consist of increased frequency of thrombotic events, as a result of tissue factor activation secondary to the exposure of the subendothelial collagen.

**Figure 15:**
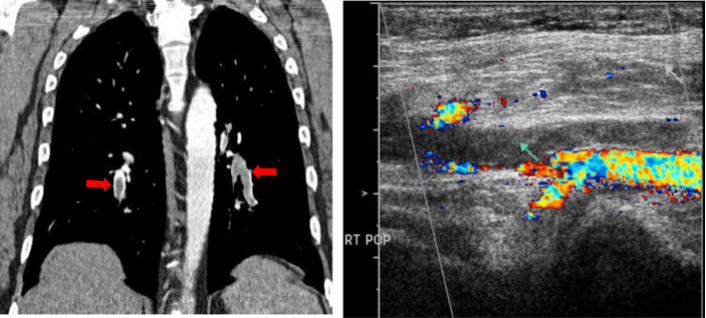
**39 year old male with glioblastoma multiformae on Bevacizumab. A**. Coronal CT demonstrates multiple bilateral pulmonary emboli (arrow). **B**. US Doppler of the right lower extremity demonstrates echogenic material completely filling the left popliteal vein (arrow) and extending to the proximal left posterior tibial vein consistent with Deep Venous Thrombosis.

In a randomized phase II study evaluating the efficacy and safety of bevacizumab in patients with previously untreated advanced colorectal cancer [61,69,70], venous thromboembolism was the most significant adverse event, together with hypertension, proteinuria, and epistaxis. Bevacizumab has been also associated with increased risk of pulmonary embolism [62,63] (Figure 15).

More recently, two small molecules blocking the VEGF receptor (VEGFR) have shown promising results in the treatment of renal cell carcinoma and gastrointestinal stromal tumors (GISTs): sorafenib (Nexavar; Bayer Pharmaceuticals Corporation, West Haven, CT), approved by the FDA in 2005 as a second-line treatment for advanced renal cell carcinoma after cytokine failure, and sunitinib (Sutent; Pfizer, Inc., New York), approved in 2006 for patients with GIST previously treated with imatinib and for advanced renal cell carcinoma as a first-line treatment. Both of these agents have been strongly associated with thromboembolic events [71].

#### Cardiac toxicity

**Figure 16:**
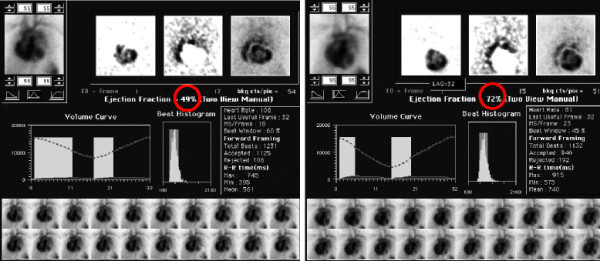
**58 year old female on trastuzumab (Herceptin). A**. MUGA scan obtained after 3 months of therapy with Herceptin shows that the EF has dropped significantly to 49% (Figure 16A). **B**. The ejection fraction recovers to 72% (Figure 16B) 1 week after discontinuation of therapy. Trastuzumab is known to cause this effect, which is worsened with concomitant anthracycline administration.

Cardiac toxicity is one major concern related to trastuzumab administration [71]. In 2002, a retrospective review of phase II and phase III trials of trastuzumab that evaluated the incidence and the characteristics of trastuzumab-associated cardiac dysfunction [72], demonstrated that 27% of patients receiving trastuzumab and doxorubicin, 13% of patients treated with trastuzumab and paclitaxel, and 3%–7% of patients undergoing therapy with trastuzumab alone developed cardiac dysfunction (Figure 16). Most of these patients were symptomatic (75%) and improved with standard treatment for CHF (79%). Since these results were published, trastuzumab combined with anthracyclines has not been recommended outside clinical trials. The pathophysiology of trastuzumab-related cardiotoxicity has not yet been completely elucidated; however, physiologic analysis revealed parameters of dilated cardiomyopathy, including chamber dilation wall thinning, and decreased contractility.

## Conclusion

New imaging modalities are available to assess treatment response. PET have the ability to assess tissue viability more precisely than CT and detect metastatic lesions that would have been missed on conventional imaging. Other functional imaging techniques, such as dynamic contrast-enhanced magnetic resonance imaging (DCE-MRI) and perfusion CT imaging, have been found to be more useful than morphological changes in the assessment of tumor response, although not frequently used in most institutions.

With the discovery and introduction of new MTT agents, the treatment for a wide variety of cancers has been revolutionized. As these treatments become increasingly recognized as therapeutic options, physicians who interpret imaging studies should be aware of different patterns of response and possible side effects of these new MTT agents.

## Competing interests

The authors declare that they have no competing interests.

## Authors’ contributions

FFS is responsible for drafting the first and subsequent versions of the manuscript, as well as editing and collection of images and figures. AS (Andrew Smith) is responsible for editing the subsequent versions of the manuscript, as well as legends of the figures. JJ is Responsible for editing the multiple subsequent versions of the manuscript. CJ is Responsible for drafting the first and subsequent versions of the manuscript, as well as editing and collection of images and figures. KO is Responsible for drafting the first and subsequent versions of the manuscript, as well as editing and collection of images and figures. CA edited the manuscript and provided figures and legends. AS (Atul Shinagare) is responsible for drafting the first and subsequent versions of the manuscript, as well as editing and collection of images and figures. Responsible for drafting the subsequent versions of the manuscript, as well as collection of images and edition of the figures. All authors read and approved the final manuscript.

## References

[B1] Skeel R Khlief S Biologic and pharmacologic basis of cancer chemotherapy and biotherapy Handbook of Cancer Chemotherapy. 7th edition. chapter 1 2007 Philadelphia: Lippincott Williams and Wilkins, a Wolters Kluwer business 1 31

[B2] Wisnivesky JP Yankelevitz D Henschke CI Stage of lung cancer in relation to its size: part 2 Evidence. Chest 2005 127 1136 1139 10.1378/chest.127.4.1136 15821186

[B3] Choi H Charnsangavej C Faria SC Macapinlac HA Burgess MA Patel SR Chen LL Podoloff DA Benjamin RS Correlation of computed tomography and positron emission tomography in patients with metastatic gastrointestinal stromal tumor treated at a single institution with imatinib mesylate: proposal of new computed tomography response criteria J Clin Oncol 2007 25 1753 1759 17470865 10.1200/JCO.2006.07.3049

[B4] Schiller JH Harrington D Belani CP Langer C Sandler A Krook J Zhu J Johnson D Comparison of four chemotherapy regimens for advanced non-small-cell lung cancer N Engl J Med 2002 346 92 98 11784875 10.1056/NEJMoa011954

[B5] Reck M Crino L Advances in anti-VEGF and anti-EGFR therapy for advanced non-small cell lung cancer Lung Cancer 2009 63 1 9 18579254 10.1016/j.lungcan.2008.05.015

[B6] Le Chevalier T Lynch T Adjuvant treatment of lung cancer: current status and potential applications of new regimens Lung Cancer 2004 46 33 39 10.1016/s0169-5002(04)80039-4 15698530

[B7] Socinski MA Adjuvant therapy of resected non-small cell lung cancer Clin Lung Cancer 2004 6 3 162 169 15555217 10.3816/CLC.2004.n.029

[B8] Choi H Response evaluation of gastrointestinal stromal tumors Oncologist 2008 13 4 7 18434631 10.1634/theoncologist.13-S2-4

[B9] Fisher ER Wang J Bryant J Fisher B Mamounas E Wolmark N Pathobiology of preoperative chemotherapy: findings from the National Surgical Adjuvant Breast and Bowel (NSABP) protocol B-18 Cancer 2002 95 681 695 12209710 10.1002/cncr.10741

[B10] Sataloff DM Mason BA Prestipino AJ Seinige UL Lieber CP Baloch Z Pathologic response to induction chemotherapy in locally advanced carcinoma of the breast: a determinant of outcome J Am Coll Surg 1995 180 297 306 7874340

[B11] Zhao B Schwartz L Larson S Imaging surrogates of tumor response to therapy: anatomic and functional biomarkers J Nucl Med 2009 50 239 249 19164218 10.2967/jnumed.108.056655

[B12] Goudarzi B Wahl R PET/CT evaluation of bronchioloalveolar carcinoma: correlation with CT and FDG/PET findings J Nucl Med 2008 49 363 10.2967/jnumed.108.052712 18794276

[B13] Choi H Charnsangavej C Faria S CT evaluation of the response of gastrointestinal stromal tumors after imatinib mesylate treatment: A quantitative analysis correlated with FDG PET findings AJR Am J Roentgenol 2004 183 1619 1628 15547201 10.2214/ajr.183.6.01831619

[B14] Li WW Tumor angiogenesis: molecular pathology, therapeutic targeting, and imaging Acad Radiol 2000 7 800 811 11048878 10.1016/s1076-6332(00)80629-7

[B15] Miles KA Hayball MP Dixon AK Functional images of hepatic perfusion obtained with dynamic CT Radiology 1993 188 405 411 8327686 10.1148/radiology.188.2.8327686

[B16] Cenic A Nabavi DG Craen RA Gelb AW Lee TY Dynamic CT measurement of cerebral blood flow: a validation study Am J Neuroradiol 1999 20 63 73 9974059

[B17] Cenic A Nabavi DG Craen RA Gelb AW Lee TY A CT method to measure hemodynamics in brain tumors: validation and application of cerebral blood flow maps Am J Neuroradiol 2000 21 462 470 10730636 PMC8174983

[B18] Hattori H Miyoshi T Okada J Yoshikawa K Arimizu N Hattori N Tumor blood flow measured using dynamic computed tomography Invest Radiol 1994 29 873 876 7852037 10.1097/00004424-199410000-00002

[B19] Gould RG Lipton MJ McNamara MT Sievers RE Koshold S Higgins CB Measurement of regional myocardial blood flow in dogs by ultrafast CT Invest Radiol 1988 23 348 353 3384614

[B20] Blomley MJ Coulden R Bufkin C Lipton MJ Dawson P Contrast bolus dynamic computed tomography for the measurement of solid organ perfusion Invest Radiol 1993 28 Suppl. 5 S72 S77 8282508 10.1097/00004424-199311001-00023

[B21] Blomley MJ Coulden R Dawson P Kormano M Donlan P Bufkin C Liver perfusion studied with ultrafast CT J Comput Assist Tomogr 1995 19 424 433 7790553 10.1097/00004728-199505000-00016

[B22] Gillard J Antoun N Burnet N Pickard J Assessment of quantitative computed tomographic cerebral perfusion imaging with positron emission tomography Neurol Res 2000 22 457 464 10935216 10.1080/01616412.2000.11740700

[B23] Wintermark M Thiran JP Maeder P Schnyder P Meuli R Simultaneous measurement of regional cerebral blood flow by perfusion CT and stable xenon CT: a validation study Am J Neuroradiol 2001 22 905 914 11337336 PMC8174953

[B24] Nabavi DG Cenic A Dool J Smith RM Espinosa F Craen RA Gelb AW Lee TY Quantitative assessment of cerebral hemodynamics using CT: stability, accuracy, and precision studies in dogs J Comput Assist Tomogr 1999 23 506 515 10433275 10.1097/00004728-199907000-00003

[B25] Gillard JH Antoun NM Burnet NG Pickard JD Reproducibility of quantitative CT perfusion imaging Br J Radiol 2001 74 552 555 11459735 10.1259/bjr.74.882.740552

[B26] Crabb SJ Patsios D Sauerbrei E Ellis PM Arnold A Goss G Leighl NB Goodman SL Pepper MS Tumor cavitation: impact on objective response evaluation in trials of angiogenesis inhibitors in non–small-cell lung cancer J Clin Oncol 2009 27 3 404 410 19047292 10.1200/JCO.2008.16.2545

[B27] Marom EM Martinez CH Truong MT Lei X Sabloff BS Munden RF Gladish GW Herbst RS Morice RC Stewart DJ Jimenez CA Blumenschein GR Jr Onn A Tumor cavitation during therapy with antiangiogenesis agents in patients with lung cancer J Thorac Oncol 2008 3 351 357 18379352 10.1097/JTO.0b013e318168c7e9

[B28] Hicks R Role of 18 F-FDG PET in assessment of response in non-small cell lung cancer J Nucl Med 2009 50 31S 42S 19380411 10.2967/jnumed.108.057216

[B29] Fujita S Mio T Sonobe M Accuracy of epidermal growth factor receptor mutation analysis on the basis of small biopsy specimens in patients with nonsmall cell lung cancer Int J Cancer 2006 119 1751 1752 16646071 10.1002/ijc.22012

[B30] Kimura H Fujiwara Y Sone T Kunitoh H Tamura T Kasahara K Nishio K High sensitivity detection of epidermal growth factor receptor mutations in the pleural effusion of non-small cell lung cancer patients Cancer Sci 2006 97 642 648 16827805 10.1111/j.1349-7006.2006.00216.x PMC11160100

[B31] Kimura H Kasahara K Kawaishi M Kunitoh H Tamura T Holloway B Detection of epidermal growth factor receptor mutations in serum as a predictor of the response to gefitinib in patients with non-small cell lung cancer Clin Cancer Res 2006 12 3915 3921 16818687 10.1158/1078-0432.CCR-05-2324

[B32] Shih JY Gow CH Yu CJ Yang CH Chang YL Tsai MF Hsu YC Chen KY Su WP Yang PC Epidermal growth factor receptor mutations in needle biopsy/aspiration samples predict response to gefitinib therapy and survival of patients with advanced nonsmall cell lung cancer Int J Cancer 2006 118 963 969 16152581 10.1002/ijc.21458

[B33] Montaudon M Latrabe V Pariente A Corneloup O Begueret H Laurent F Factors influencing accuracy of CT-guided percutaneous biopsies of pulmonary lesions Eur Radiol 2004 14 1234 1240 14963689 10.1007/s00330-004-2250-3

[B34] Vahid B Marik P Pulmonary complication of novel antineoplastic agents for solid tumors Chest 2008 133 528 538 18252919 10.1378/chest.07-0851

[B35] Meyers JL Katzenstein AA, Askin FB Pathology of drug-induced lung disease Katzenstein and Askin’s surgical pathology of non-neoplastic lung disease, Volume 4 1997 3 Philadelphia, Pa: Saunders 81 111

[B36] Pietra GG Pathologic mechanisms of drug-induced lung disorders J Thorac Imaging 1991 6 1 7 1990154 10.1097/00005382-199101000-00003

[B37] Kay JM Hasleton PS Drug-induced lung disease Spencer’s Pathology of the Lung 1996 5 New York, NY: McGraw-Hill 551 595

[B38] Inoue A Saijo Y Maemondo M Severe acute interstitial pneumonia and gefitinib Lancet 2003 361 137 139 12531582 10.1016/S0140-6736(03)12190-3

[B39] Inoue A Saijo Y Maemondo M Gomi K Tokue Y Kimura Y Diffuse alveolar damage after ZD 1839 therapy in a patient with non-small cell lung cancer Lung Cancer 2003 40 339 342 12781434 10.1016/s0169-5002(03)00043-6

[B40] Ohyanagi F Ando Y Nagashima F Narabayashi M Sasaki Y Acute gefitinib-induced pneumonitis Int J Clin Oncol 2004 9 406 409 15549594 10.1007/s10147-004-0418-0

[B41] Sumpter K Harper-Wynne C O'Brien M Congleton J Severe acute interstitial pneumonia and gefitinib Lung Cancer 2004 43 367 368 15165098 10.1016/j.lungcan.2003.09.016

[B42] Ieki R Saitoh E Shibuya M Acute lung injury as a possible adverse drug reaction related to gefitinib Eur Respir J 2003 22 179 181 12882469 10.1183/09031936.03.00098503

[B43] Nagaria NC Cogswell J Choe JK Kasimis B Side effects and good effects from new chemotherapeutic agents: Gefitinib-induced interstitial fibrosis J Clin Oncol 2005 23 2423 2424 15800334 10.1200/JCO.2005.04.055

[B44] Vahid B Esmaili A Erlotinib-associated acute pneumonitis: report of two cases Can Respir J 2007 14 167 170 17464382 10.1155/2007/832605 PMC2676839

[B45] Vahid B Mehrotra A Trastuzumab (Herceptin)-associated lung injury Respirology 2006 11 655 658 16916343 10.1111/j.1440-1843.2006.00907.x

[B46] Radzikowska E Szczepulska E Chabowski M Bestry I Organising pneumonia caused by trastuzumab (Herceptin) therapy for breast cancer Eur Respir J 2003 21 552 555 12662016 10.1183/09031936.03.00035502

[B47] Tripathy D Slamon DJ Cobleigh M Arnold A Saleh M Mortimer JE Murphy M Stewart SJ Safety of treatment of metastatic breast cancer with trastuzumab beyond disease progression J Clin Oncol 2004 22 1063 1070 15020607 10.1200/JCO.2004.06.557

[B48] Clamon G Herndon J Kern J Govindan R Garst J Watson D Green M Lack of trastuzumab activity in nonsmall cell lung carcinoma with overexpression of erb-B2:39810: a phase II trial of Cancer and Leukemia Group B Cancer 2005 103 1670 1675 15751020 10.1002/cncr.20950

[B49] Romond EH Perez EA Bryant J Suman VJ Geyer CE Jr Davidson NE Tan-Chiu E Martino S Paik S Kaufman PA Trastuzumab plus adjuvant chemotherapy for operable HER2-positive breast cancer N Engl J Med 2005 353 1673 1684 16236738 10.1056/NEJMoa052122

[B50] Duran I Siu LL Oza AM Chung TB Sturgeon J Townsley CA Characterization of the lung toxicity of the cell cycle inhibitor temsirolimus Eur J Cancer 2006 42 1875 1880 16806903 10.1016/j.ejca.2006.03.015

[B51] Atkins MB Hidalgo M Stadler WM Logan TF Dutcher JP Hudes GR Park Y Liou SH Marshall B Boni JP Dukart G Sherman ML Randomized phase II study of multiple dose levels of CCI-779, a novel mammalian target of rapamycin kinase inhibitor, in patients with advanced refractory renal cell carcinoma J Clin Oncol 2004 22 909 918 14990647 10.1200/JCO.2004.08.185

[B52] Rothenburger M Teerling E Bruch C Lehmkuhl H Suwelack B Bara C Wichter T Hinder F Schmid C Stypmann J SO: **Calcinurin inhibitor-free immunosuppression using everolimus (certican) in maintenance heart transplant recipients: 6 months follow-up** J Heart Lung Transplant 2007 26 250 257 17346627 10.1016/j.healun.2007.01.017

[B53] Jullien V Perrin C Peyrade F Lemoigne F Chichmanian RM Blaive B Alveolar hypoxemic interstitial pneumonia related to rituximab therapy Rev Mal Respir 2004 21 407 410 15211254 10.1016/s0761-8425(04)71304-2

[B54] Burton C Kaczmarski R Jan-Mohamed R Interstitial pneumonitis related to rituximab therapy N Engl J Med 2003 348 2690 2691 12826649 10.1056/NEJM200306263482619

[B55] Kanelli S Ansell SM Habermann TM Inwards DJ Tuinstra N Witzig TE Rituximab toxicity in patients with peripheral blood malignant B-cell lymphocytosis Leuk Lymphoma 2001 42 1329 1337 11911416 10.3109/10428190109097760

[B56] Swords R Power D Fay M O'Donnell R Murphy PT Interstitial pneumonitis following rituximab therapy for immune thrombocytopenic purpura (ITP) Am J Hematol 2004 77 103 104 15307117 10.1002/ajh.20135

[B57] Cooper JA Jr White DA Matthay RA Drug-induced pulmonary disease. 1. Cytotoxic drugs Am Rev Resp Dis 1986 133 321 340 3511808 10.1164/arrd.1986.133.2.321

[B58] Erasmus JJ McAdams HP Rossi SE High-resolution CT of drug-induced lung disease Radiol Clin North Am 2002 40 1 61 72 11813820 10.1016/s0033-8389(03)00109-x

[B59] McAdams HP Rosado-de-Christenson ML Wehunt WD Fishback NF The alphabet soup revisited: the chronic interstitial pneumonias in the 1990s Radiographics 1996 16 1009 1033 8888388 10.1148/radiographics.16.5.8888388

[B60] Bonadonna G Valagussa P Brambilla C Primary chemotherapy in operable breast cancer: eight-year experience at the Milan Cancer Institute J Clin Oncol 1998 16 93 100 9440728 10.1200/JCO.1998.16.1.93

[B61] Johnson DH Fehrenbacher L Novotny WF Herbst RS Nemunaitis JJ Jablons DM Langer CJ DeVore RF Randomized phase II trial comparing bevacizumab plus carboplatin and paclitaxel with carboplatin and paclitaxel alone in previously untreated locally advanced or metastatic non-small cell lung cancer J Clin Oncol 2004 22 2184 2191 15169807 10.1200/JCO.2004.11.022

[B62] Sandler A Gray R Perry MC Brahmer J Schiller JH Dowlati A Lilenbaum R Johnson DH Paclitaxel-carboplatin alone or with bevacizumab for non-small-cell lung cancer N Engl J Med 2006 355 2542 2550 17167137 10.1056/NEJMoa061884

[B63] Herbst RS Sandler AB Non-small cell lung cancer and antiangiogenic therapy: what can be expected of bevacizumab? Oncologist 2004 9 19 26 15178812 10.1634/theoncologist.9-suppl_1-19

[B64] Demetri GD von Mehren M Blanke CD Van den Abbeele AD Eisenberg: **Efficacy and safety of imatinib mesylate in advanced gastrointestinal stromal tumors** N Engl J Med 2002 347 472 480 12181401 10.1056/NEJMoa020461

[B65] Talpaz M Shah NP Kantarjian H Donato N Nicoll J Paquette R Cortes J O'Brien S Nicaise C Bleickardt E Blackwood-Chirchir MA Dasatinib in imatinib-resistant Philadelphia chromosome-positive leukemias N Engl J Med 2006 354 24 2531 2541 16775234 10.1056/NEJMoa055229

[B66] Kantarjian H Pasquini R Hamerschlak N Dasatinib or high-dose imatinib for chronic-phase chronic myeloid leukemia after failure of first-line imatinib: a randomized phase 2 trial Blood 2007 109 12 5143 5150 17317857 10.1182/blood-2006-11-056028

[B67] Kantarjian H Sawyers C Hochhaus A Guilhot F Schiffer C Gambacorti-Passerini L Hematologic and cytogenetic responses to imatinib mesylate in chronic myelogenous leukaemia N Engl J Med 2002 346 645 652 11870241 10.1056/NEJMoa011573

[B68] Goldsby R Pulsipher M Adams R Coffin C Albritton K Wagner L Unexpected pleural effusions in 3 pediatric patients treated with STI-571 J Pediatr Hematol Oncol 2002 24 694 695 12439048 10.1097/00043426-200211000-00020

[B69] Kabbinavar F Hurwitz HI Fehrenbacher L Meropol NJ Novotny WF Phase II, randomized trial comparing bevacizumab plus fluorouracil (FU)/leucovorin (LV) with FU/LV alone in patients with metastatic colorectal cancer J Clin Oncol 2003 21 60 65 12506171 10.1200/JCO.2003.10.066

[B70] Midgley R Kerr D Bevacizumab—current status and future directions Ann Oncol 2005 16 999 1004 15939715 10.1093/annonc/mdi208

[B71] Widakowich C de Castro G Jr de Azambuja E Dinh P Side effects of approved molecular targeted therapies in solid cancers Oncologist 2007 12 1443 1455 18165622 10.1634/theoncologist.12-12-1443

[B72] Seidman A Hudis C Pierri MK Shak S Paton V Ashby M Murphy M Stewart SJ Keefe D Cardiac dysfunction in the trastuzumab clinical trials experience J Clin Oncol 2002 20 1215 1221 11870163 10.1200/JCO.2002.20.5.1215

